# Performance Evaluation of Machine Learning Frameworks for Aphasia Assessment

**DOI:** 10.3390/s21082582

**Published:** 2021-04-07

**Authors:** Seedahmed S. Mahmoud, Akshay Kumar, Youcun Li, Yiting Tang, Qiang Fang

**Affiliations:** Department of Biomedical Engineering, College of Engineering, Shantou University, Shantou 515041, China; akshay.kumar@student.rmit.edu.au (A.K.); 19ycli12@stu.edu.cn (Y.L.); 17yttang@stu.edu.cn (Y.T.)

**Keywords:** aphasia assessment, deep neural network, machine learning framework, Mandarin, speech impairment

## Abstract

Speech assessment is an essential part of the rehabilitation procedure for patients with aphasia (PWA). It is a comprehensive and time-consuming process that aims to discriminate between healthy individuals and aphasic patients, determine the type of aphasia syndrome, and determine the patients’ impairment severity levels (these are referred to here as aphasia assessment tasks). Hence, the automation of aphasia assessment tasks is essential. In this study, the performance of three automatic speech assessment models based on the speech *dataset-type* was investigated. Three types of datasets were used: healthy subjects’ dataset, aphasic patients’ dataset, and a combination of healthy and aphasic datasets. Two machine learning (ML)-based frameworks, classical machine learning (CML) and deep neural network (DNN), were considered in the design of the proposed speech assessment models. In this paper, the DNN-based framework was based on a convolutional neural network (CNN). Direct or indirect transformation of these models to achieve the aphasia assessment tasks was investigated. Comparative performance results for each of the speech assessment models showed that quadrature-based high-resolution time-frequency images with a CNN framework outperformed all the CML frameworks over the three dataset-types. The CNN-based framework reported an accuracy of 99.23 ± 0.003% with the healthy individuals’ dataset and 67.78 ± 0.047% with the aphasic patients’ dataset. Moreover, direct or transformed relationships between the proposed speech assessment models and the aphasia assessment tasks are attainable, given a suitable dataset-type, a reasonably sized dataset, and appropriate decision logic in the ML framework.

## 1. Introduction

Speech assessment is used extensively in the diagnosis of Parkinson’s and aphasia diseases [1,2,3,4,5,6,7,8,9,10,11,12,13,14]. Aphasia is an acquired neurogenic language disorder that can be evaluated with one of the well-known assessment tools, such as the Chinese Rehabilitation Research Center Aphasia Examination (CRRCAE [15], for Chinese-dialect-speaking patients), the Aachen Aphasia Test (AAT [16], for German-speaking patients) and the Boston Diagnostic Aphasia Examination (BDAE [17], for English-speaking patients). These tests are used by a skilled speech-language pathologist (SLP) to assess people with aphasia (PWA). Commonly, there are three aphasia assessment tasks whereby an SLP performs a comprehensive examination of the patient’s communication abilities, including speaking, expressing ideas, understanding language, reading, and writing. These tasks are the discrimination between normal and aphasic speech [9], the assessment of the degree of severity of impairment for aphasic patients [10,12], and the classification of aphasia syndromes (such as Global aphasia, Broca’s aphasia, Wernicke’s aphasia and amnesic aphasia) [13,14]. Conventional methods of aphasia assessment and rehabilitation are resource-intensive processes that require the presence of an SLP. Therefore, the automation of the aphasia assessment process is essential.

Most research on automatic aphasia assessment [5,9,10,12,13,14,18] has focused on a single aphasia assessment task using automatic speech recognition techniques and a machine learning (ML) framework with a fixed *dataset-type*. Generally, three types of training datasets are used for aphasia assessment tasks: (1) healthy subjects’ dataset, (2) aphasic patients’ dataset, or (3) a combination of healthy and aphasic dataset [10,12] (hereafter, these datasets are referred to as *dataset-type*. Description and investigation of the performance of the ML-based frameworks for each aphasia assessment task is essential to guide the rapid implementation of a framework, and to assist in planning the training dataset collection protocol, by which the most suitable training *dataset-type* can be identified. Moreover, a generalized ML framework that allows the flexible transformation between aphasia assessment tasks is required. In this paper, three speech assessment models based on the training *dataset-type* were explored and derived from the general ML framework. The direct or transform relationship between these models and the aphasia assessment tasks are presented. The comparative performance of various classifiers within each proposed speech assessment models is presented and discussed.

This paper is organized as follows. Section 2 presents the related research on aphasia and ML-based methods employed in the literature for automatic aphasic speech assessment. Section 3 presents the materials and methods and includes details about the data collection, the CML framework, the convolutional neural networks (CNN) and the performance evaluation metrics. In Section 4 and Section 5, the results and a discussion of the findings are presented, respectively.

## 2. Related Work

Artificial intelligence (AI) algorithms are increasingly being used in automatic aphasia assessment. For example, in [18], researchers investigated eight machine learning (ML) classifiers’ performance on two aphasia assessment tasks. In one of the tasks, they used naming datasets to discriminate between healthy individuals and aphasic patients, while in the other task, the PatLight aphasia dataset was used to recognize the patients’ aphasic syndromes. The findings showed that none of the ML classifiers performed well with all datasets. Also, they suggested that the selection of the classifier is task-dependent. In [13], Kohlschein et al. proposed an ML-based multi-class automatic aphasia assessment system to classify various aphasia syndromes. Their classification model achieved a low accuracy of 44.3%, and they suggested that a large dataset was required to improve the classification performance. According to the literature, the problem of the automatic multi-class classification of various types of aphasia syndrome is complex and nontrivial. This type of aphasia assessment task requires a large dataset from each class/aphasia syndrome.

The majority of aphasia research uses speech utterance for aphasia diagnosis and assessment. However, some researchers have used a neuroimaging dataset instead of a speech dataset to diagnose aphasia [19,20,21]. In [20], Kristinsson et al. used ML techniques to predict aphasia severity and specific language measures based on a multimodal neuroimaging dataset. The dataset included task-based functional magnetic resonance imaging (fMRI), diffusion-based fractional anisotropy (FA)-values, cerebral blood flow (CBF), and lesion-load data. Their findings showed that different neuroimaging modalities carry complementary information that can be integrated to depict how brain damage and the remaining functionality of intact brain tissue translate into language function in aphasia. Other aphasia assessment tasks such as the discrimination between normal and aphasic speech and the discrimination between various aphasia syndromes were not investigated in their research. Matias-Guiu et al. used a non-speech utterance dataset and proposed machine learning algorithms with a language test dataset to predict five variants of primary progressive aphasia (PPA) [21]. Further, they used their previously proposed cluster-based classification algorithm with the FDG positron emission tomography (PET) regional metabolism to classify patients into the five variants of PPA. They evaluated the performance of several classifiers and found that the instance-based learning (IBK) classifier had the best performance with a true positive rate of 0.931. Overall, this study applied classical machine learning algorithms to classify various types of PPA. Other types of aphasia assessment tasks were not investigated in their work. Researchers in [10] proposed an automatic speech lucidity assessment approach for Mandarin-speaking aphasic patients using a deep learning-based technique. The method in [10] established a relationship between the severity level of aphasic patients’ speech and three speech lucidity features (articulation, fluency, and tone). Their research focused on one of the aphasia assessments tasks, which assesses the severity of impairment for an aphasic patient. To achieve other aphasia assessment tasks, a ML framework transformation is required.

The efficacy of ML-based aphasia assessment methods depends on the quality and size of the datasets, the selection of a ML framework (whether it is a classical machine learning (CML)-based or a deep neural network (DNN)-based framework), the selection of an appropriate classifier, and the choice of a suitable training dataset. The training dataset-type of an ML-based aphasia assessment model determines whether the assessment task is to discriminate between normal and aphasic speech, assess the severity degree of speech impairment for aphasic patients, or classify the syndrome of aphasia impairment [18]. In this paper, three speech assessment models based on the training dataset-type were explored and derived from the general ML framework. Based on aphasia assessment tasks, the ML framework and a suitable training dataset-type were decided. The comparative performance investigation for various classifiers and the CNN-based classification method within each proposed speech assessment models is presented and discussed.

## 3. Materials and Methods

### 3.1. Dataset

In [10], we collected Mandarin datasets from healthy subjects and aphasic patients. In this study, we utilized the same datasets as used in [10]. The detailed information on the experiment setup, participants’ clinical information, and data acquisition can be found in [10]. However, for the sake of coherence, the crucial details about the experiment have been included here. The datasets used in this study are listed below:

1. Six Mandarin-vowels, ten Mandarin-nouns, and ten Mandarin-verbs. This dataset was collected from thirty-four healthy subjects (11 females, mean age: 21.5 ± 3.1 years), considering the CRRCAE standard [10,15].
2. Six Mandarin-vowels, ten Mandarin-nouns, and ten Mandarin-verbs. This dataset was collected from twelve aphasic patients (mean age: 61.8 ± 14.4 years), considering the CRRCAE standard [10,15].

The twelve aphasic patients were recruited from the First Affiliated Hospital of Shantou University, Guangdong province, China, and the Second Hospital of Jiaxing, Zhejiang province, China (Table 1). The data collection procedure was approved by the Ethics Committees of both hospitals, and the data collection protocol was strictly followed to ensure the investigation complied with the Declaration of Helsinki [10]. In addition to the aphasic dataset, thirty-four healthy subjects were recruited from Shantou University (STU), China. The healthy and aphasic dataset-type were used to investigate the performance of the three speech assessment models and identify a suitable dataset-type for each aphasia assessment task.

In this research, the collected speech from all healthy subjects and aphasic patients were Mandarin-vowels, isolated Mandarin words (i.e., *huo3*, which means fire), and combined Mandarin words (i.e., *lou2 fang2*, which means building). The six Mandarin-vowels considered in this paper are: ā, ō, ē, ī, ū, and ǖ [11]. Twenty keywords were (ten nouns and ten verbs) considered in this research, which were taken from the CRRCAE standard [15]. The selected keywords belong to everyday objects, food, and activities categories; these are listed in [10]. Each word was repeated for an average of three times per aphasic patient and five times per healthy participant. Five of the twelve patients had recorded vowels only because they could not record words during the data collection.

The speech of healthy participants and aphasic patients was recorded using a Lenovo B613 voice recording pen with a sampling rate of 48 kHz. The voice recorder has low-noise and high-fidelity sound. Healthy participants’ speech data were recorded at STU in a vacant office space where all external noise sources were eliminated. The recording environment of the patients (9 out of 12 patients) from the Second Hospital of Jiaxing was a professionally designed speech therapy room with soundproof walls. The other three patients’ speech was recorded in a vacant ward located in a quiet corner of the corridor in the First Affiliated Hospital of STU. The data collection environment for the healthy subjects and patients was largely consistent [10]. Furthermore, the speech data were manually inspected, and all outliers (4% of the total dataset) were removed [10].

### 3.2. Machine Learning Frameworks

A typical automatic speech assessment method deploys either a classical machine learning (CML)-based framework or a deep neural network (DNN)-based framework. In this study, the CML and the DNN-based frameworks were investigated to select the optimum framework for each aphasia assessment task mentioned above. The three speech assessment models were derived from the CML and the DNN-based frameworks. These models differ in the type of training and testing dataset applied to them and the *dataset-type*, while they maintain the same classification structure:

1. Model-A: training and testing of the ML frameworks with healthy datasets using a 5-fold cross-validation method.
2. Model-B: training and testing of the ML frameworks with aphasic patients’ datasets using a 5-fold cross-validation method.
3. Model-C: training of the ML framework with healthy datasets and testing with the aphasic patients’ datasets.

Model A, B and C are hereafter referred to as speech assessment models. These models can be directly or indirectly suitable for aphasia assessment tasks.

#### 3.2.1. Classical Machine Learning Framework

**Figure 1:**
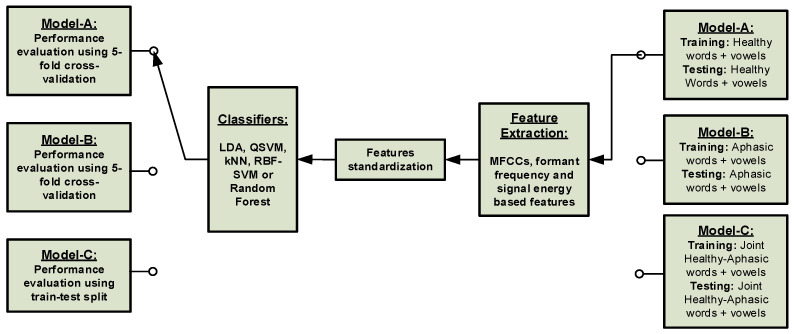
A typical classical machine learning framework for the three speech assessment models.

The classical machine learning (CML) framework for automatic speech recognition (ASR) consists of a speech feature extraction stage, a classifier and/or a decision stage that assigns the classifier output to a class of one of the Mandarin vowels: ā, ō, ē, ī, ū and ǖ or one of the twenty Mandarin keywords. Three speech assessment models based on the training *dataset-type* were investigated thoroughly, and their association with each of the aphasia assessment tasks was determined. Figure 1 shows a typical CML-based framework for the three speech assessment models, Model-A, Model-B and Model-C.

Conventional ASR features were used in the feature stage of the three speech assessment models. A set of 51 ASR features based on Mel-Frequency Cepstral Coefficients (MFCCs), energy signals and the formant frequencies were calculated for the proposed models [20,21,22,23,24]. The feature vectors of all data samples were standardized. The standardization was performed by subtracting the mean and dividing by the standard deviation of each of the data sample’s feature vector. The feature standardization is essential in CML for reducing the effect of a feature dominating others due to its large magnitude.

In this research, several classifiers within the CML framework were evaluated for each speech assessment model. The classifiers considered with the three models were:

1. Quadratic support vector machine (QSVM)
2. A radial basis function (RBF) kernel SVM
3. Linear discriminant analysis (LDA)
4. Random forest
5. K-nearest neighbours (kNN).

The choice of the CML’s classifiers in this paper was influenced by the comparative performance investigation reported in [19,25], which found that the random forest (RF) and a radial basis function (RBF) kernel SVM outperformed 14 other classification algorithms. However, other classification algorithms such as fuzzy and neuro-fuzzy-based techniques can be used when the data is affected by uncertainty and/or inaccuracies [26,27,28].

The performance of different classifiers for each speech assessment model were compared. Two separate datasets were constructed from each of the *dataset-types* (healthy, aphasic patients or combination): one containing all vowels and words speech data (26 classes) (hereafter named the *vowels + words* dataset) and the other containing only words speech data (20 classes) (hereafter named the *only-words* dataset). A five-fold cross-validation was used to estimate the five classifiers’ performance in Model-A and Model-B, while a train-test split method was used to estimate the classifiers’ performance in Model-C. The five-fold cross-validation was used to overcome the data overfitting problem and generalize the prediction. For each of the five folds, a model is trained using four of the folds as the training data, while the resulting model is validated on the remaining part of the *dataset-type*.

#### 3.2.2. Deep Neural Network Framework

**Figure 2:**
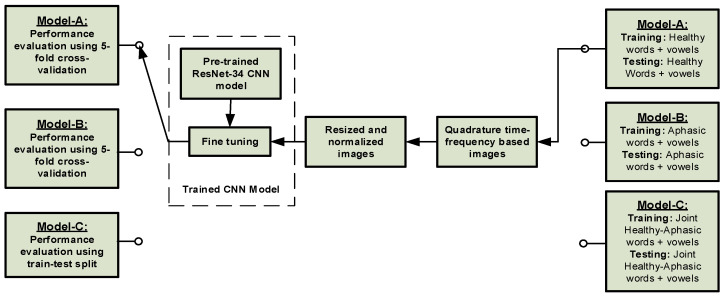
A typical convolutional neural network (CNN)-based classification framework for the three speech assessment models.

Besides the CML framework, a convolutional neural network (CNN) with a high-resolution time-frequency image was discussed to design the three speech assessment models. The CNN models are widely used across various applications and domains, including aphasia assessment tasks [9]. Furthermore, a comparative study of the CML framework and the CNN framework is presented. In this research, the hyperbolic T-distribution (HTD) [29,30] was used as a time-frequency-based image input to the CNN model within each model. The HTD has been found to produce a high-resolution TF image of Mandarin speech signals; hence, it the speech signal classification can be improved using the CNN model [10,31,32,33]. Figure 2 shows a typical CNN-based classification framework for the three-speech assessment models, Model-A, Model-B and Model-C. Figure 2 demonstrates the pre-training process and the performance evaluation method for the three-speech assessment models.

Due to the unavailability of a large speech dataset, transfer learning (TL) was utilized for training the CNN model in this paper. The ResNet-34 pre-trained CNN model was employed, which was fine-tuned with healthy participants’ speech or patients’ speech time-frequency distribution (TFD) images based on the choice of classification models shown in Figure 2 [34]. Two separate models for the two datasets: the *vowels + words* dataset (26 classes) and the *only-words* dataset (20 classes), were trained for each classification models. In order to utilize the weights of the pre-trained ResNet-34 model for transfer learning, all TFD RGB color images were resized to 224 × 224 × 3 pixels and normalized as per ImageNet dataset characteristics, before feeding them to the pre-trained ResNet-34 CNN model [34]. Cyclical learning rates with a maximum learning rate of 0.03 was used to fine-tune the pre-trained model [35]. A learning rate range test was used to estimate the maximum learning rate’s optimum value [35]. ADAM optimizer with default parameters for β1 = 0.9 and β2 = 0.999 was used for training, with a cross-entropy loss function [36]. To prevent overfitting, weight decay was utilized with a multiplying factor of 0.01, which was chosen empirically [37]. A five-fold cross-validation was adopted to estimate the performance of the classifier in classifying the two datasets for Model-A and Model-B, while a train-test split method was used for Model-C. The CNN model for the classification of the *vowels + words* dataset was trained for 20 epochs, and the model for the classification of the *only-words* dataset was trained for 15 epochs. A batch size of 128 was used for both datasets. All models were trained on NVIDIA Tesla P40 GPU in fastai, a PyTorch-based deep neural networks library [38].

**Figure 3:**
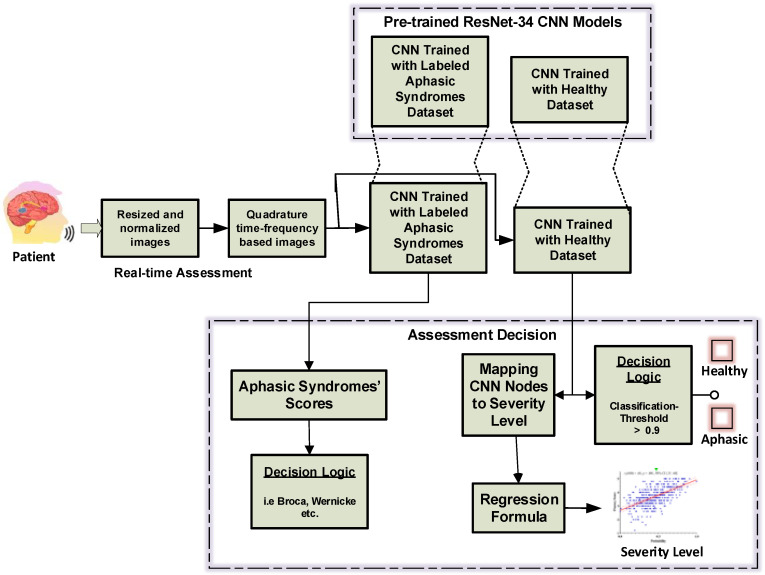
A general framework for aphasia assessment tasks.

The proposed CNN-based framework transformation of the three-speech assessment models, which are depicted in Figure 2, to achieve the three aphasia assessment tasks is shown in Figure 3. The direct and indirect transformation of the three speech models and the aphasia assessment tasks will be discussed further in the Section 5.

#### 3.2.3. Decision on Classifiers’ Output

Assigning a classifier’s output in an ML framework to correct classes/categories requires a decision logic as a final stage. Logics in the decision stage can range from a simple scoring method, as in binary classification, to complex mapping and regression algorithms. This stage in the ML framework is of utmost significance in the aphasia assessment tasks. An example of a decision logic for the binary classification, such as the discrimination between normal and aphasic speech, is given by the pseudocode in Algorithm 1.

**Algorithm 1.** Decision Logic for Binary Classification*//This process below usually uses the classifier’s output* *//Binary Classifier has Single Output Node* **1: Start** **2: *κ*  ⃪  Classification Threshold (cut-off)** **3: C1  ⃪  Normal Class** **4: C2  ⃪  Aphasia Class** **5: Q  ⃪  Classifier output**//*normalized between 0 and 1* **6: if Q > *κ*** then **C1**  //*the tested speech is normal* else **C2**  //*the tested speech is aphasic* **7: End**

#### 3.2.4. Performance Evaluation Metrics

The performance of the ML framework, CML and DNN, can be measured using various performance indicators (PI). The performance indicators considered in this research to evaluate the selected ML frameworks over healthy and aphasic speech were accuracy, precision, and recall. In this paper, the average value and the standard deviation were calculated for each PI from the cross-validation results.

Accuracy measures the percentage of correctly classified speech samples. The total classification accuracy was evaluated by

(1) $$\mathrm{Accuracy}=100\cdot \frac{\left(TP+TN\right)}{\left(TP+TN+FP+FN\right)}\%$$

where *TP* is the number of true positive classification, *TN* is the number of true negative classification, *FP* is the false-positive classification, and *FN* is the number of false-negative classification.

Precision was evaluated by dividing the true positives by the sum of true positives and false positives, while recall was obtained by dividing the true positives by the sum of true positives and true negatives.

(2) $$\mathrm{Precision}=100\cdot \frac{TP}{\left(TP+\;FP\right)}\%$$

(3) $$\mathrm{Recall}=100\cdot \frac{TP}{\left(TP+\;FN\right)}\%$$

## 4. Results

The selection and implementation of an appropriate ML framework for aphasia assessment tasks depend on the *dataset-type*. The purpose of aphasia assessment tasks is; the discrimination between normal and aphasic speech [9], the assessment of the degree of severity of the impairment for an aphasic patient [10] and the classification of different aphasia syndromes [13]. This section presents the comparative performance results for the three ML models based on the training and testing datasets. The results compare various classifiers, including DNN within each model (Model-A, Model-B and Model-C). The direct or transform relationships between the proposed speech assessment models and the aphasia assessment tasks will be discussed in the Section 5. The summary of the results for each model is presented in the following subsections.

### 4.1. ML Performance on Healthy Dataset: Model-A

In this section, the performance of CML and the CNN on the healthy subjects’ dataset, Model-A, is presented. In Model-A, two ML frameworks, CML and CNN, were considered and compared. The CML-based framework has five different classifiers.

**Figure 4:**
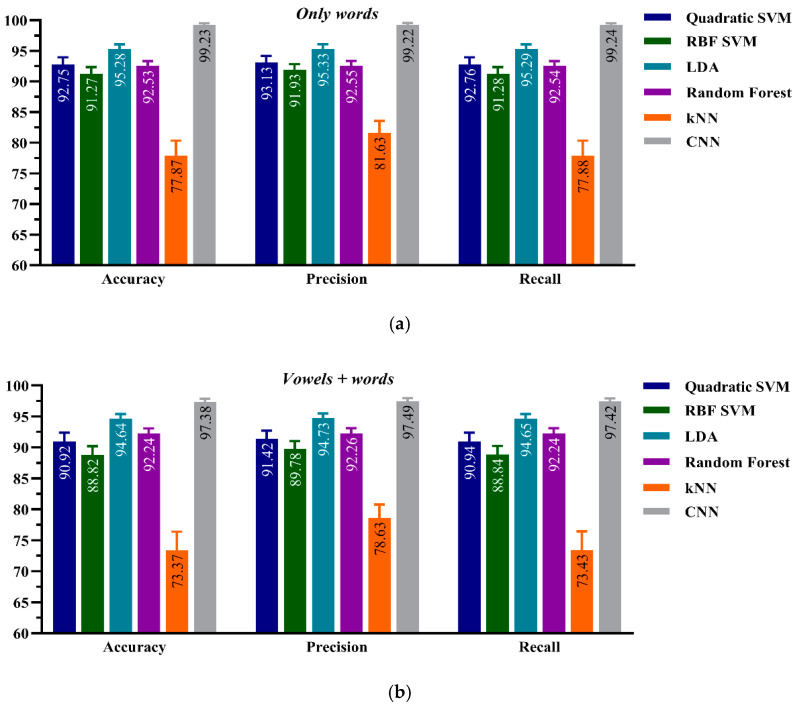
Performance evaluation for the CNN and classical machine learning (CML) classification frameworks, Model-A, on (**a**) the *Only words* (20 classes) of healthy *dataset-type* and (**b**) the *Vowels + Words* (26 classes) of healthy *dataset-type.*

The performance results for the two frameworks are compared in Figure 4. The results show that the ResNet-34 CNN framework with the HTD TF images as input-based classification outperformed all CML algorithms employed to classify the two datasets in terms of the three performance evaluation metrics. Among the CML classification algorithms, the LDA algorithm outperformed the other classifier algorithms. The CNN model has a higher accuracy of 99.23 ± 0.003% for the *only-words* dataset compared to 95.28 ± 0.79% for the LDA with the same dataset. Also, with the larger class size of 26, the CNN model has 97.39 ± 0.004% accuracy, which is higher than that of the other CML algorithms. Moreover, all classifiers except the kNN performed well on this dataset and their accuracies exceeded 90%.

### 4.2. ML Performance on Aphasic Dataset: Model-B

This section presents the performance of CML and the CNN frameworks on the aphasic patients’ dataset, Model-B.

**Figure 5:**
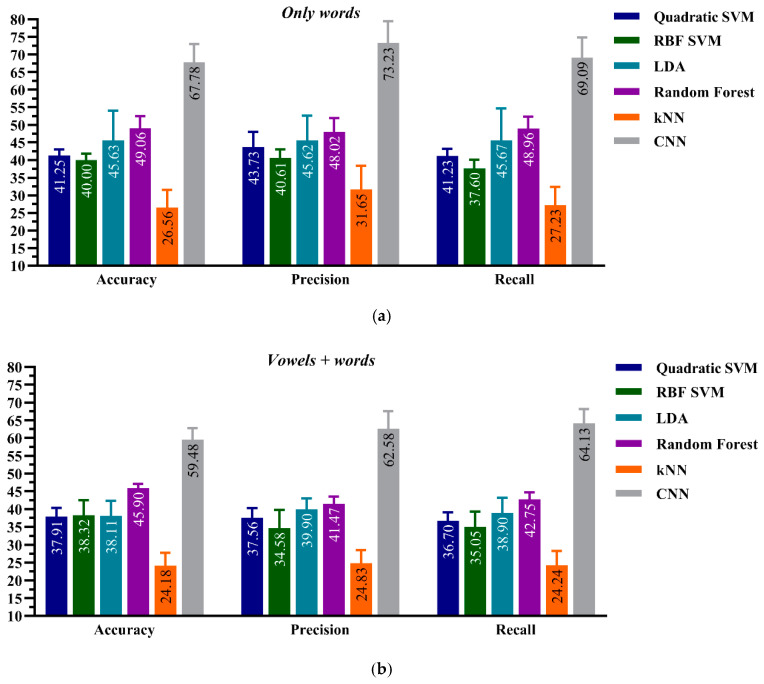
Performance evaluation for the CNN and CML classification frameworks, Model-B, on (**a**) the *Only words* of aphasic patients’ *dataset-type* and (**b**) the *Vowels + Words* of aphasic patients’ *dataset-type.*

The performance evaluation of this model, in terms of accuracy, precision, and recall, is shown in Figure 5. Similar to Model-A, the comparative results showed that the ResNet-34 CNN model with the HTD TF images as input-based classification outperformed all CML algorithms employed to classify the two datasets (the *vowels + words* and the *only-words* datasets). However, in Model-B, the random forest classifier outperformed the other CML classifiers. The CNN model has a higher accuracy of 67.78 ± 0.047% for the *only-words* dataset compared with 49.06 ± 1.91% for the random forest classifier with the same dataset. All ML algorithms, including CNN, have significantly low performance on the aphasic patients’ dataset. The Model-B results show that the deviations from the evaluation metrics are higher than those in Model-A, as shown in Figure 3 and Figure 4. This variability around the mean values is due to the wide range of impairment severity levels in the recruited patients. 

### 4.3. ML Performance onr Joint Healthy-Aphasic Datasets: Model-C

This section presents the performance of CML and the DNN frameworks on the joint healthy-aphasic dataset, Model-C. Various CML classifiers and DNN were used with this dataset.

**Figure 6:**
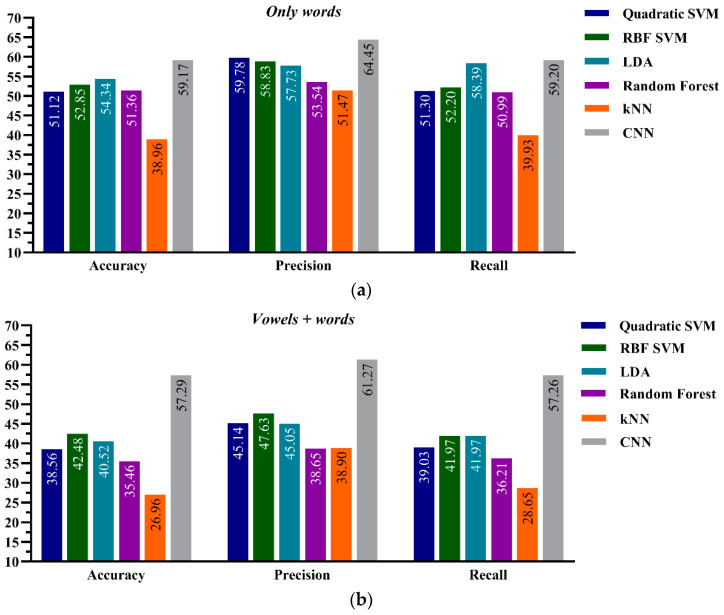
Performance evaluation for the CNN and CML classification frameworks, Model-C, on (**a**) the *Only words* of aphasic patients and healthy subjects’ *dataset-types* and (**b**) the *Vowels + Words* of aphasic patients and healthy subjects’ *dataset-types*.

The performance evaluation for this model is shown in Figure 6. Similarly, the comparative performance results show that the ResNet-34 CNN model with the HTD TF images as input-based classification outperformed all CML classifiers employed to classify the two datasets. Among the CML classifiers, the results in this figure show that the LDA algorithm outperformed the other CML algorithms with the *only-words* datasets. In contrast, the RBF SVM algorithm outperformed the other CML classifiers with the *vowels + words* datasets. Like Model-B, all machine learning algorithms, including CNN, have significantly poor performance on the joint healthy-aphasic dataset-type. In both datasets, CNN reported greater than 50% accuracy. Specifically, the CNN has 59.17% accuracy with the *only-words* dataset and 57.29% accuracy with the *vowels + words* dataset.

## 5. Discussion

Several tasks and processes are proposed in the literature to assess and rehabilitate a patient with aphasia (PWA) [9,10,13]. However, in the literature, most research focuses on a single aphasia assessment task with little discussion on the flexibility of the general ML framework for other assessment tasks and the relationship with the *dataset-type*. In other words, what is the optimum ML framework and the most suitable training *dataset-types* that can be used to discriminate between normal and aphasic patients, to assess the severity degree of impairment for aphasic patients [10,12] and to classify aphasia syndromes?

In this paper, the results show that the *dataset-type*, whether for healthy subjects, aphasic patients, or the joint healthy and aphasia dataset, has an enormous impact on the performance of all ML frameworks. Furthermore, the comparative results show that the CNN-based framework outperformed all CML frameworks over the three speech assessment models (Model-A, B and C). The findings for each of the models and their relation to each aphasia assessment tasks are discussed in the following subsections.

### 5.1. Healthy Dataset: Model-A

In this section, the healthy subjects’ dataset-type was used to train and test the ML frameworks, CML and CNN. The ResNet-34 CNN model with the HTD TF images as input outperformed all CML algorithms employed to classify the two datasets, *vowels + words* and *only-words* datasets within each *dataset-type*. The performance of CML with automatic speech recognition (ASR)-based features depends on the quality of the extracted features and their ability to readily separate between classes. On the other hand, the CNN-based classification model can extract unique features from the high-resolution images; hence, it gives better classification results than the CML [10,31,32].

This type of speech assessment model is a multi-class classification problem that classifies 20 Mandarin words and 6 Mandarin vowels. The model was found to be suitable to investigate the performance of various CML classifiers, including the CNN-based framework, and can also be used to study these model’s suitability for different automatic speech recognition (ASR) applications. It should be noted that the class imbalance problem in the input dataset can be handled with random minority oversampling or the cost-sensitive resampling approach.

To investigate the possibility of transforming this model for any of the aphasia assessment tasks, the decision logic and the dataset-type are crucial. Firstly, for assessing the severity degree of impairment for an aphasic patient, this model can be turned into a regression problem by mapping the classifiers’ output to the severity levels’ ground truth as reported in [10]. Hence, the only change needed for Model-A to achieve this task is to include a mapping processor at the decision stage. Besides, the real-time testing dataset should include healthy and aphasic speech.

Secondly, for the assessment task of discriminating between normal and aphasic speech [6,7], Model-A can either be retrained to include an aphasic dataset to act as a binary classification problem or the model remains the same. For this task, the real-time input dataset should include healthy and aphasic speech. As shown in Algorithm 1 and Figure 3, a binary decision logic is required at the classifier’s output. When testing with the healthy and aphasic datasets in this paper, the transformation of this model to a binary classifier to discriminate between normal and aphasic speech, achieved 100% accuracy even when the classification cut-off threshold, κ, at the decision stage was set to 0.9 (output probability at the classifier’s output node). This can be proved by the results for Model-A and Model-C, wherein Model-A, when testing with the healthy dataset, the accuracy was reported by the CNN as 99.23 ± 0.003%, while for the same model when tested by the aphasic dataset, the best accuracy was 59.17%.

Finally, this model is inappropriate for the assessment task of discrimination between various aphasia syndromes since it was only trained with a healthy dataset. A large dataset for each aphasia syndrome is required during the training process to achieve the task of discriminating between various aphasia syndromes.

### 5.2. Aphasic Dataset: Model-B

The aphasic patients’ dataset was used to train and test the ML frameworks in this section. Similarly, the CNN-based framework with the HTD TF images as input, outperformed the CML framework with various classifiers in classifying the two datasets. Both ML frameworks have a significantly poor performance on the aphasic patients’ dataset in both *vowels + words* and *only-words* datasets. The degradation in the performance of this model is due to the diversity of the aphasic dataset [10]. The recruited patients have different speech impairment severity levels, as reported in [10], that resulted in complex and unresolvable common features. Also, the aphasic multi-types (i.e., Global aphasia, Broca’s aphasia, Wernicke’s aphasia and Amnesic aphasia) speech datasets are scarce and often have a small sample for each severity level group [10]. This finding agrees with what was reported in the literature where data scarcity [39], abnormal speech patterns [40], and speaker variability [41] are challenging to any classification problem.

In its current form, Model-B is not suitable for any aphasia assessment tasks since it was trained with aphasic speech only. However, it can be retrained with properly labeled data that include all aphasia syndromes such as Global aphasia, Broca’s aphasia, Wernicke’s aphasia and amnesic aphasia datasets to discriminate between them [13]. In this aphasia assessment task, the performance of this model depends on the size of the training dataset from each aphasia type, which is a real challenge due to domain data scarcity [39]. With a large dataset for each aphasia type, classifiers can create distinct boundaries in the features space, resulting in high classification accuracy. Similarly, the CNN model will perform better with a large labeled dataset since it has better generalization in terms of feature extraction from high-resolution images [10].

### 5.3. Joint Healthy-Aphasic Dataset: Model-C

The joint healthy-aphasic *dataset-type* was used to train and test the ML frameworks. The CNN framework outperformed other CML frameworks employed to classify the two datasets. This model is similar to Model-A with the only difference being the type of the testing dataset. Therefore, a ready transformation between the two models is attainable.

This model has low overall performance accuracy when tested with aphasic speech [10]. In other words, patients’ impairment severity levels were not labeled in the training datasets. Hence, when speech samples of healthy subjects were fed to the model, the CNN-based framework could successfully classify the speech sample with 99.23 ± 0.003% accuracy. Conversely, if aphasic speech samples were fed to the same model, it would classify the aphasic speech with a low probability, depending on the severity level. As reported in [10], the CNN model’s final node activations are highly correlated with patients’ impairment severity levels. For example, when two of the recruited patients [10] with different impairment severity levels spoke the Mandarin verb *chuan1 yi1*, the CNN’s output activation at the true class node (named the normalized true-class output activation (TCOA) in [10]) was 0.35 for the patient with the high severity level, and it was 0.73 for the patient with the low severity level. The wide range of patients’ severity levels resulted in low overall accuracy in this model. This finding makes this model suitable to discriminate between normal and aphasic speech, as presented in the Model-A discussion. Model-A can be readily transformed into Model-C (see Figure 3) to achieve similar aphasia assessment tasks performed by Model-A.

In conclusion, the results show that the CNN-based framework is the optimum framework compared to the CML-based framework. It should be noted that the CNN-based framework is more complex and requires more computation resources than the CML-based framework, because the CNN-based framework uses a ResNet-34 CNN model. However, as the proposed automatic aphasia assessment frameworks use offline analysis, it is noted that the framework complexity and computation resource requirement are not a constraint. Moreover, to achieve automatic aphasia assessment tasks, two models are required. One model could be either A or B, while the other model should be trained with a large dataset of aphasia syndromes.

## 6. Conclusions and Future Work

In this paper, the performance of three automatic speech assessment models based on *dataset-type* has been investigated. Speech data recorded from twelve aphasic patients and thirty-four healthy subjects, including six Mandarin vowels and twenty different Mandarin words, formed three *dataset-types*, which were used in this research. Two ML-based frameworks, classical machine learning (CML) and convolutional neural network (CNN), were considered to design the proposed speech assessment models. The results showed that the CNN framework outperforms the CML-based framework in automatic speech assessment for all three *dataset-types*. Furthermore, we discussed that the relationship and transformation between the proposed speech assessment models and the aphasia assessment tasks are achievable by providing a suitable *dataset-type*, a reasonably sized dataset, and appropriate decision logic at the ML framework. Finally, this study presented a general framework for aphasia assessment tasks, which can serve as an aphasia assessment guide for dataset collection, experimental design, and classification framework selection.

In the future, due to the scarcity of the aphasia syndrome *dataset-type*, data collection for this domain is required to improve the accuracy of the CNN-based assessment and discrimination of aphasia syndromes. Also, the implementation of a CNN framework capable of performing the three-aphasia assessment tasks is required. The proposed method should also be evaluated for solving other problems, such as assessing the limb mobility of stroke patients.

## Figures and Tables

**Table 1:** Aphasic patients’ details.

Number of Patients	Gender Male/Female	Age, Yrs. (Mean ± SD)	Cardinal Symptom (#)	Native Dialect (#)
12	7/5	61.8 ± 14.4	Broca (6)	Mandarin (6)
			Dysarthria (3)	
			Anomic (1)	Teochew (2)
			Combined (1)	
			Transcortical motor (1)	Jiaxing (4)

## Data Availability

The data presented in this study and the implementation source code are available on request from the corresponding authors.
